# Interferon alpha regulates MAPK and STAT1 pathways in human hepatoma cells

**DOI:** 10.1186/1743-422X-8-157

**Published:** 2011-04-06

**Authors:** Lan-Juan Zhao, Xian Hua, Sheng-Fei He, Hao Ren, Zhong-Tian Qi

**Affiliations:** 1 Department of Microbiology, Shanghai Key Laboratory of Medical Biodefense, Second Military Medical University, Shanghai, PR China

## Abstract

**Background:**

Signaling events triggered by interferon (IFN) account for the molecular mechanisms of antiviral effect. JAK-STAT pathway plays a critical role in IFN signaling, and other pathways are also implicated in IFN-mediated antiviral effect. Changes in mitogen-activated protein kinase (MAPK) and STAT1 pathways were evaluated in human hepatoma cells Huh7 and HepG2 upon IFN alpha treatment.

**Results:**

Phosphorylation of ERK was significantly and specifically up-regulated, whereas enhanced phosphorylation of upstream kinase MEK was unobservable upon IFN alpha treatment. A mild increase in p38 MAPK, SAPK/JNK and downstream target ATF-2 phosphorylation was detectable after exposure to IFN alpha, indicating differential up-regulation of the MAPK signaling cascades. Moreover, STAT1 phosphorylation was strongly enhanced by IFN alpha.

**Conclusion:**

IFN alpha up-regulates MAPK and STAT1 pathways in human hepatoma cells, and may provide useful information for understanding the IFN signaling.

## Background

Interferon (IFN), the first cytokine discovered in 1957 [1], has been studied extensively and advances have been made in biochemical and molecular mechanisms underlying production and action of IFN system. IFN family is classified as types I, II, and III, and plays a pivotal role in innate defense toward virus infection. Being the first line of defense, type I IFN including IFN alpha (IFN α) and IFN beta, exert their potent antiviral activities immediately after virus infection by inhibiting viral replication and enhancing immune responses [2-4].

Signaling events triggered by IFN are involved in the molecular mechanisms of the antiviral effects. In particular, JAK-STAT pathway plays a critical role in the signaling events induced by IFN, and this pathway is initiated by IFN through interaction with cell surface specific receptors [5-7]. Upon IFN binding to its receptor, the receptor-associated tyrosine kinases JAK are phosphorylated and activated, the kinases subsequently activate transcription factors STAT, and the activated STAT translocate to nuclear followed by binding to IFN-stimulated response elements and modulating transcription of IFN-stimulated genes [8]. The products of IFN-stimulated genes are known to be responsible for the antiviral effects of IFN [9]. Apart from the JAK-STAT pathway, a number of pathways may also be important for the IFN-dependent biological responses. Mitogen-activated protein kinase (MAPK) signaling pathways are composed of three subfamilies including ERK, SAPK/JNK, and p38 MAPK, and activation of MAPK pathways contributes to some human diseases [10]. MAPK pathways are activated by a wide variety of extracellular signals such as stress, growth factors, and cytokines. The p38 MAPK or ERK is rapidly phosphorylated and activated in response to IFN α treatment [11,12]. However, involvement of MAPK pathways in the IFN signaling needs to be fully elucidated.

Hepatitis C virus (HCV) infection is the major cause of human liver diseases. IFN α is the current approved treatment for HCV infection [13]. We reported that the MAPK signaling pathways triggered by HCV envelope protein E2 were involved in the viral pathogenesis [14-16]. Since certain signaling events triggered by IFN α account for its antiviral effect, we wondered whether the MAPK pathways were also affected and an association between MAPK and STAT1 pathways under IFN α treatment. Difference in cellular response was evaluated after exposure to IFN α. Human hepatoma cells are usually used as models for evaluation of the IFN signaling. Thus, the aim of this study is to reveal regulation of MAPK and STAT1 signaling pathways by IFN α in human hepatoma cells Huh7 and HepG2.

## Results

### 1. Influence of IFN α on MEK

**Figure 1:**
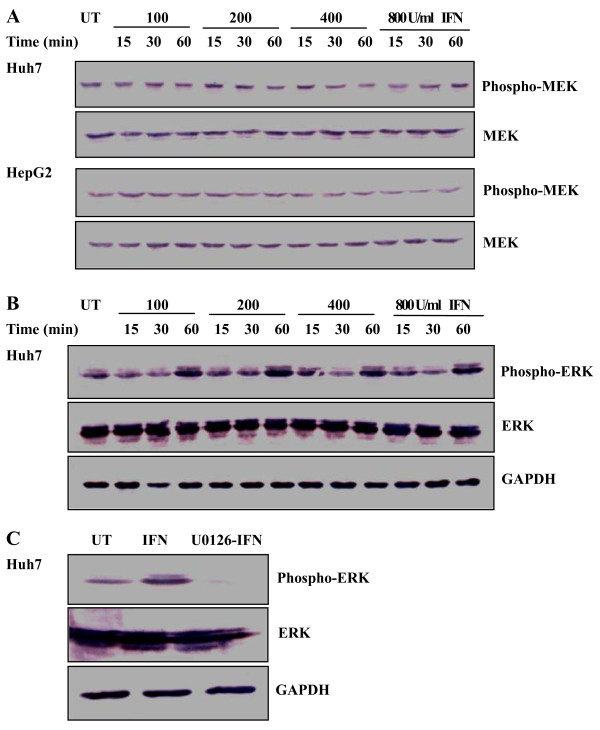
**Changes in MEK and ERK phosphorylation in response to IFN**. Huh7 and HepG2 cells were treated with the indicated concentrations of IFN α for the indicated time periods. Phosphorylated and total MEK were analyzed in the cells by Western blotting using antibodies against phospho-MEK or MEK (A). Huh7 cells were treated with the indicated concentrations of IFN α for the indicated time periods. Phosphorylated and total ERK were analyzed using antibodies against phospho-ERK or ERK (B). Huh7 cells were pretreated with U0126 before addition of IFN α. Phosphorylated and total ERK were analyzed (C). GAPDH was determined as a control for protein loading. UT, untreated. One representative experiment out of three is shown.

Activation of mitogen-activated protein kinases occurs through phosphorylation of threonine and tyrosine. Phosphorylation levels of kinases are usually evaluated to estimate MAPK pathways in response to stimuli [17-19]. To examine whether MEK-ERK pathway would be affected under IFN treatment, changes in MEK phosphorylation were initially analyzed by Western blotting. Huh7 and HepG2 cells were treated with the increasing concentrations of IFN α (0, 100, 200, 400, 800 U/ml) for the indicated time course (15, 30, 60 min) and MEK phosphorylation was analyzed before and after the treatment. Figure 1A showed that level of MEK phosphorylation after the IFN α treatment was similar to that before such treatment in Huh7 and HepG2 cells. For long time treatment of IFN, Huh7 cells were cultured in the medium containing the 800 U/ml IFN α for 12, 24, 48, or 72 hrs. Compared with basal level of MEK phosphorylation without the IFN treatment, there was no increase in MEK phosphorylation with the IFN treatment for 12 or 24 hrs and the kinase phosphorylation was reduced following the treatment for 48 or 72 hrs (Figure 4). Amount of total MEK, independent of phosphorylation, was monitored as a control for protein loading. IFN α seems to be no enhanced effect on MEK phosphorylation.

### 2. Prevention of IFN α-induced ERK activation by U0126

Next, changes in downstream kinase ERK phosphorylation were estimated in Huh7 cells treated with the IFN α. As shown in Figure 1B, phosphorylation of ERK was increased after exposure to the IFN. Upon the treatment with IFN α at concentrations ranging from 100 to 800 U/ml, level of ERK phosphorylation was high after 15 min treatment compared to the treatment for 30 min, and a significant increase in ERK phosphorylation was observed at 60 min. In addition, MEK inhibitor U0126 was applied to estimate specific regulation of the kinase cascade by the IFN α. Huh7 was pretreated with 10 μM U0126 prior to the addition of 100 U/ml IFN α. Figure 1C showed that level of ERK phosphorylation was markedly decreased in the cells pretreated with the U0126, indicating that pretreatment with U0126 prevents the IFN α-induced ERK phosphorylation. Constant level of total ERK was also determined. GAPDH was determined as a control for protein loading. Thus, IFN α significantly and specifically up-regulates ERK phosphorylation.

### 3. Regulation of p38 MAPK, SAPK/JNK, and ATF-2 by IFN α

**Figure 2:**
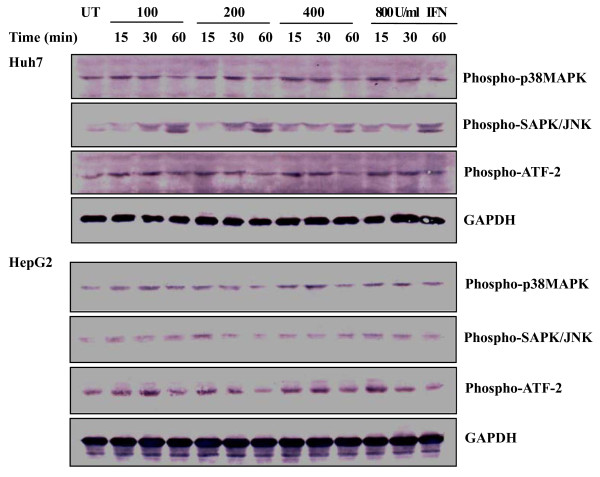
**Changes in p38 MAPK, SAPK/JNK, and ATF-2 phosphorylation in response to IFN**. Huh7 and HepG2 cells were treated with the indicated concentrations of IFN α for the indicated time periods. Phosphorylated kinases were analyzed by Western blotting using antibodies against phospho-SAPK/JNK, phospho-ATF-2, or phospho-p38 MAPK mAb. GAPDH was determined as a control for protein loading. UT, untreated. Similar results were obtained in four independent experiments, and one representative experiment is shown

To evaluate effects of IFN on p38 MAPK and SAPK/JNK, cell lysates from Huh7 and HepG2 treated with the different concentrations of IFN α at the indicated time points were examined by Western blot analysis. In comparison with phosphorylation of p38 MAPK without the IFN α treatment, level of p38 MAPK phosphorylation was slightly increased following the IFN α treatment (Figure 2). As for SAPK/JNK, differential profiles were observable in response to the IFN α treatment between HepG2 and Huh7. In HepG2 cells, the IFN α had no obvious effect on SAPK/JNK phosphorylation. Whereas SAPK/JNK phosphorylation was enhanced by the IFN α in a time dependent-manner in Huh7 cells, and the IFN α treatment for 60 min led to a significant increase in SAPK/JNK phosphorylation. GAPDH was determined as a control for protein loading.

Transcription factor ATF-2 is a downstream target of p38 MAPK and SAPK/JNK pathways. SAPK/JNK and p38 MAPK are known to phosphorylate and activate ATF-2. Since the upstream kinases p38 MAPK and SAPK/JNK were activated by the IFN α, changes in ATF-2 phosphorylation were thereby assessed. Figure 2 showed that the IFN α treatment caused a slight increase in ATF-2 phosphorylation. Interestingly, treatment of Huh7 with the IFN for 15 min seemed to be suitable for ATF-2 phosphorylation, which was consistent with the p38 MAPK phosphorylation. Thus, p38 MAPK, SAPK/JNK, and ATF-2 are differentially activated by IFN α.

### 4. Regulation of STAT1 by IFN α

**Figure 3:**
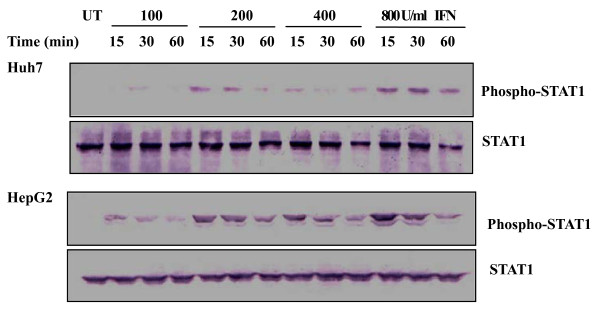
**Changes in STAT1 phosphorylation in response to IFN**. Huh7 and HepG2 cells were treated with the indicated concentrations of IFN α for the indicated time periods. Western blotting was performed to analyze phosphorylated and total STAT1 using antibody against phospho-STAT1 or STAT1. UT, untreated. Experiments were performed three times with similar results, and representative results are shown.

**Figure 4:**
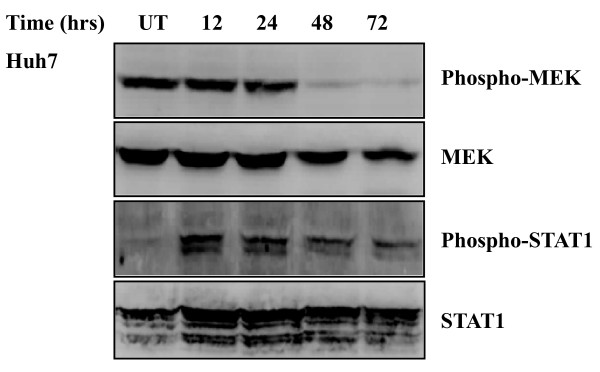
**Kinetic changes in MEK and STAT1 phosphorylation after exposure to IFN**. Huh7 cells were cultured in the DMEM containing the IFN α for 12, 24, 48, and 72 hrs, respectively. Western blotting was performed to analyze phosphorylated and total MEK and STAT1. UT, untreated. One representative experiment out of five is shown.

Activation of STAT1 is involved in the classical IFN signaling pathways. Huh7 and HepG2 cells were treated with the increasing concentrations of IFN α for the indicated time course and phosphorylation of STAT1 was examined in the cells by Western blot analysis. Figure 3 showed that level of STAT1 phosphorylation was increased in response to the IFN α treatment, and such phosphorylation was enhanced by the IFN α in a concentration dependent-manner, as evidenced that 800 U/ml IFN α treatment resulted in strong phosphorylation of STAT1 compared with the treatment of IFN α at the other concentrations. Moreover, phosphorylation of STAT1 was also increased upon the IFN α treatment for a long time period. In Huh7 cells, level of STAT1 phosphorylation was high following the 800 U/ml IFN α treatment for 12 or 24 hrs compared to the treatment for 48 or 72 hrs (Figure 4). As a control for protein loading, blots were probed with antibodies against STAT1. The data show that IFN α is responsible for the elevated STAT1 phosphorylation.

## Discussion

Being the effective therapy for HCV infection, IFN α has been focused to reveal the molecular mechanisms underlying antiviral effect. Among the multiple mechanisms, modulation of the signaling events by IFN α is particularly important for the antiviral effect. In this report, we have investigated regulation of the MAPK and STAT1 signaling pathways by IFN α in human hepatoma cells. Our data show that the phosphorylation of ERK, p38 MAPK, SAPK/JNK, and ATF-2 is up-regulated by IFN α, whereas IFN α seems to be no enhanced effect on the MEK phosphorylation. In response to IFN α treatment, the STAT1 phosphorylation is also enhanced. The profiles of kinetic kinase phosphorylation are shown.

IFN is believed to exert its antiviral effect predominantly through JAK-STAT signaling pathway. IFN sequentially phosphorylates and activates JAK kinases and STAT transcription factors to stimulate the transcription of IFN-stimulated genes. STAT family has seven known members, and STAT1 and STAT2 play central roles in induction of the IFN-dependent antiviral state [9]. Studies show that some viruses have evolved strategies to evade the host immune responses and set up successful infections by targeting STAT pathway. Ebola virus, rotavirus, Venezuelan equine encephalitis virus, Sindbis virus, and Marburg virus are shown to prevent the nuclear translocation and phosphorylation of STAT1, resulting in the impairment of the IFN α-dependent antiviral effect [20-23]. As for HCV, the interference of HCV proteins with IFN α-induced JAK-STAT pathway has been proposed to be an escape strategy of HCV [24]. For instance, HCV core protein inhibits IFN α-induced activation of STAT1 in hepatic cells [25]. IFN α-mediated STAT activation is blocked in Huh7 cells containing the HCV genomic replicon [26]. HCV nonstructural protein 5A inhibits IFN α signaling through suppression of STAT1 phosphorylation in hepatocyte-derived cells [27]. Consistent with these findings, here we document that the STAT1 phosphorylation is strongly enhanced by IFN α in human hepatoma cells. Our data show that the phosphorylation of STAT1 is enhanced upon the IFN α treatment not only for a short time period but also for a long time period. These results are confirmed by using the IFN α-2a and 2b.

Changes in the MAPK signaling pathways induced by the HCV proteins contribute to HCV pathogenesis. In this regards, it is interesting to examine whether MAPK pathways are also interfered by IFN α and the association between MAPK pathways and JAK-STAT pathway triggered by IFN α. In the present study, we addressed the regulation of MEK, ERK, p38 MAPK, SAPK/JNK, and ATF-2 by the IFN α. The cellular response was evaluated upon the different concentrations and time periods of the IFN α treatment. The ERK pathway is activated by growth factors, cytokines, and virus infection. Interestingly, we found that the phosphorylation of ERK was significantly and specifically up-regulated by the IFN α, whereas enhanced phosphorylation of upstream kinase MEK was unobservable. The p38 MAPK and SAPK/JNK are the stress-related signal transduction pathways. The IFN α treatment led to the mild increase in p38 MAPK and SAPK/JNK phosphorylation, and also resulted in the enhancement of downstream target ATF-2 phosphorylation. Our data indicate that IFN α is capable of differentially up-regulating MAPK pathways in human hepatoma cells. Under the same condition of IFN α treatment, Huh7 appears to be susceptible to the IFN α compared with HepG2, implying that the regulation of MAPK pathways by IFN α may depend on cell types. In support of our data, reports show that the p38 MAPK, JNK, and ERK are activated by IFN gamma or the type I IFN [28-30]. Thus, in addition to classical JAK-STAT pathway, MAPK pathways are also activated by IFN α and involved in the IFN signaling. Recent studies illustrate an important role for the IFN signaling pathways in triggering the host antiviral responses to HCV infection [31-34]. Our results suggest that the up-regulation of MAPK and STAT1 pathways by IFN α might be implicated in its antiviral effect, although we have not ruled out the possible involvement of other pathways in the signaling events induced by IFN α. Further studies are still necessary to determine the downstream effects of these phosphorylation events, including the double-stranded RNA-activated protein kinase, 2'-5' oligoadenylate synthetase, and Mx.

In conclusion, our results demonstrate that IFN α up-regulates MAPK and STAT1 signaling pathways in human hepatoma cells, and provide useful information for understanding the IFN signaling events.

## Methods

### 1. Materials

Recombinant human IFN α-2a and 2b were obtained from PBL Interferon Source (Piscataway, NJ) and Huaxin High Biotechnology (Shanghai, China), respectively. Alkaline phosphatase-conjugated goat anti-rabbit or anti-mouse IgG were purchased from Vector Lab (Burlingame, CA). Horseradish peroxidase-conjugated goat anti-rabbit IgG was from Invitrogen (Camarillo, CA). Chemiluminescent detection reagents were from Millipore (Billerica, MA). 5-bromo-4-chloro-3-indolyl phosphate and nitroblue tetrazolium were obtained from Sigma (St. Louis, MO). MEK inhibitor U0126 and antibodies specific for MEK, ERK, STAT1, phospho-MEK (Ser217/221), phospho-ERK (Thr202/Tyr204), phospho-SAPK/JNK (Thr183/Tyr185), phospho-p38 MAPK (Thr180/Tyr182), phospho-ATF-2 (Thr71), or phospho-STAT1 (Tyr701) were purchased from Cell Signaling Technology (Beverly, MA). Dulbecco's modified Eagle's medium (DMEM) and fetal bovine serum were from HyClone.

### 2. IFN treatment

Human hepatoma cells Huh7 and HepG2 were grown in DMEM plus 10% fetal bovine serum, 2 mM L-glutamine, 100 U/ml penicillin, and 100 μg/ml streptomycin at 37°C with 5% CO_2_. Cells at 80% confluence were washed twice with phosphate-buffered saline and then maintained for 24 hrs in serum-free DMEM before treatment with IFN α-2b. For concentration- and time-dependent stimulation of IFN, cells serum-starved were treated with increasing concentrations of IFN α-2b dissolved in 0.1% bovine serum albumin (0, 100, 200, 400, 800 U/ml) for 15, 30, or 60 min. For long time treatment of IFN, Huh7 cells were serum starved for 12 hrs and then cultured in serum-free DMEM containing 800 U/ml IFN α-2a for 12, 24, 48, and 72 hrs, respectively. Following the above treatments, cells were washed with phosphate-buffered saline, collected by centrifugation at 2,000 rpm for 5 min, lysed in sodium dodecyl sulfate sample buffer on ice, and heated for 5 min at 100°C. Cell lysates were centrifuged at 12,000 rpm for 10 min to remove cellular debris, mixed with sample loading buffer, boiled, and that equal amounts of protein extracts were subjected to Western blot analysis.

### 3. U0126 pretreatment

Huh7 cells serum-starved were pretreated for 1 h with 10 μM U0126 dissolved in dimethylsulfoxide prior to treatment with 100 U/ml IFN α-2b for another 1 h. Cells with or without the U0126 pretreatment were harvested and lysed for measurement of ERK by Western blotting.

### 4. Western blotting

Phosphorylated and total levels of signal molecules were assayed by Western blotting following the manufacturer's protocol with some modification. In brief, cell lysates were loaded onto 10% sodium dodecyl sulfate-polyacrylamide gel electrophoresis. Proteins were separated and transferred onto nitrocellulose membranes. After blocking in 5% nonfat milk for 1 h, membranes were incubated overnight at 4°C with antibodies against MEK, ERK, STAT1, phospho-MEK, phospho-ERK, phospho-SAPK/JNK, phospho-p38 MAPK, phospho-ATF-2, or phospho-STAT1 at the recommended dilutions, followed by incubation with alkaline phosphatase or horseradish peroxidase-conjugated secondary antibodies. The blots were developed with 5-bromo-4-chloro-3-indolyl phosphate and nitroblue tetrazolium substrates or chemiluminescent detection reagents. Glyceraldehyde-3-phosphate dehydrogease (GAPDH) was determined as a control for protein loading.

## Competing interests

The authors declare that they have no competing interests.

## Authors' contributions

LJZ designed and performed research, analyzed and interpreted data, and wrote the manuscript. XH, SFH, and HR performed research. ZTQ has given final approval for the version to be published. All authors read and approved the final manuscript.
